# Penumbra Detection With Oxygen Extraction Fraction Using Magnetic Susceptibility in Patients With Acute Ischemic Stroke

**DOI:** 10.3389/fneur.2022.752450

**Published:** 2022-02-11

**Authors:** Yuto Uchida, Hirohito Kan, Hiroyasu Inoue, Masahiro Oomura, Haruto Shibata, Yuya Kano, Tomoyuki Kuno, Toshihiko Usami, Koji Takada, Kentaro Yamada, Kohsuke Kudo, Noriyuki Matsukawa

**Affiliations:** ^1^Department of Neurology, Nagoya City University, Nagoya, Japan; ^2^Department of Neurology, Toyokawa City Hospital, Aichi, Japan; ^3^Department of Integrated Health Sciences, Nagoya University, Nagoya, Japan; ^4^Department of Neurology, Nagoya City East Medical Center, Nagoya, Japan; ^5^Department of Diagnostic Imaging, Hokkaido University, Hokkaido, Japan; ^6^Global Center for Biomedical Science and Engineering, Faculty of Medicine, Hokkaido University, Hokkaido, Japan

**Keywords:** acute ischemic stroke, magnetic resonance imaging, oxygen extraction fraction, penumbra, quantitative susceptibility mapping

## Abstract

**Background:**

The oxygen extraction fraction (OEF) has been applied to identify ischemic penumbral tissue, but is difficult to use in an urgent care setting. This study aimed to investigate whether an OEF map generated via magnetic resonance quantitative susceptibility mapping (QSM) could help identify the ischemic penumbra in patients with acute ischemic stroke.

**Materials and Methods:**

This prospective imaging study included 21 patients with large anterior circulation vessel occlusion who were admitted <24 h after stroke onset and 21 age-matched healthy controls. We identified the ischemic penumbra as the region with a Tmax of >6 s during dynamic susceptibility contrast-magnetic resonance imaging (DSC-MRI) and calculated the perfusion-core mismatch ratio between the ischemic penumbra and infarct core volumes. The OEF values were measured based on magnetic susceptibility differences between the venous structures and brain tissues using rapid QSM acquisition. Volumes with increased OEF values were compared to the ischemic penumbra volumes using an anatomical template.

**Results:**

Eleven patients had a perfusion-core mismatch ratio of ≥1.8, and reperfusion therapy was recommended. In these patients, the volumes with increased OEF values of >51.5%, which was defined using the anterior circulation territory OEF values from the 21 healthy controls, were positively correlated with the ischemic penumbra volumes (*r* = 0.636, 95% CI: 0.059 to 0.895, *P* = 0.035) and inversely correlated with the 30-day change in the National Institutes of Health Stroke Scale scores (*r* = −0.624, 95% CI: −0.891 to −0.039, *P* = 0.041).

**Conclusion:**

Tissue volumes with increased OEF values could predict ischemic penumbra volumes based on DSC-MRI, highlighting the potential of the QSM-derived OEF map as a penumbra biomarker to guide treatment selection in patients with acute ischemic stroke.

## Introduction

Various neuroimaging biomarkers have been applied to identify ischemic penumbral tissues (1). The oxygen extraction fraction (OEF) is an effective metric to evaluate metabolic reserve in acute ischemia (2, 3). The oxygen-15 positron emission tomography (^15^O-PET) technique is considered the gold standard for penumbra detection (4–6), although it is difficult to perform for patients with acute ischemic stroke. A magnetic resonance imaging (MRI)-based mismatch concept has been validated as a simpler and more widely applicable modality (7–9), and the difference between perfusion-weighted imaging (PWI) and diffusion-weighted imaging (DWI) can reflect physiological or pathophysiological conditions of penumbral tissue properties (10, 11). However, this procedure cannot be used for all patients and in all situations because it requires a dynamic susceptibility contrast (DSC) protocol, which involves a dynamic scan using gadolinium-containing contrast media.

Advances in MRI post-processing technology have yielded a new approach to the OEF calculation (12). Quantitative susceptibility mapping (QSM) enables direct OEF estimation by measuring venous concentrations of paramagnetic deoxygenated hemoglobin (13–15). Furthermore, the principles of echo shifting with a train of observations (PRESTO) sequence with a conventional 3.0 T MRI system can provide a rapid acquisition for magnetic susceptibility (16, 17), which would be useful in an urgent care setting.

Here, we report the first comparative analysis of ischemic penumbra based on DSC-MRI and the MRI-based OEF estimation in patients with acute ischemic stroke. We hypothesized that ischemic penumbra volumes determined using the DWI-PWI mismatch would be correlated with those with increased OEF derived from PRESTO-QSM. This study aimed to establish a method for creating an MRI-based OEF map that helps identify penumbral tissues in acute ischemic stroke.

## Materials and Methods

### Subjects

This single-center observational cohort study consecutively included 21 patients (12 men and 9 women; mean age ± SD: 76.1 ± 7.4 years), each with a large anterior circulation vessel occlusion, who were admitted <24 h after stroke onset to the Department of Neurology, Toyokawa City Hospital between March 2020 and September 2020. The patients' clinical characteristics were investigated and included the premorbid modified Rankin Scale, the National Institutes of Health Stroke Scale (NIHSS) score at arrival, time from stroke onset to MRI, and occluded vessels. Advertisements on local bulletin boards were used to recruit 21 age-matched healthy controls (11 men and 10 women; mean age ± SD: 75.3 ± 7.8 years). All participants fulfilled the following inclusion criteria: the premorbid modified Rankin Scale of <3, <90 years old, and available results from detailed neurological and comprehensive MRI examinations. Patients were excluded if they had a history of stroke or renal impairment. Clinical and radiological outcomes were assessed using follow-up MRI findings as well as the modified Rankin Scale and NIHSS scores at 30 days. This study was approved by the Institutional Review Board of Toyokawa City Hospital and written informed consent was obtained from all participants.

### Imaging Acquisition

The MRI scans were performed for each subject using a 3.0 T MRI system (Ingenia; Philips Healthcare, Best, the Netherlands) that was equipped with a 32-channel head coil. The DWI was acquired using single shot spin-echo echoplanar imaging (EPI) with the following parameters: echo-time (TE) = 73 ms, repetition time (TR) = 4,100 ms, flip angle = 90°, b = 0 and 1,000 s/mm^2^, pixel size = 2.5 × 2.5 mm^2^, slice thickness = 4 mm, 25 slices, and field of view (FOV) = 224 × 224 mm^2^. At admission and after 30 days, fluid-attenuated inversion recovery (FLAIR) and T2^*^-weighted images were acquired to identify irreversible infarctions and intracranial hemorrhages. The data for QSM were obtained using the PRESTO sequence with the following parameters: TE = 30 ms, TR = 18 ms, flip angle = 15°, parallel imaging factor = 2, FOV = 192 × 192 × 148 mm^3^, voxel size = 1.5 × 1.5 × 1.5 mm^3^, and scan time = 79 s. The PRESTO sequence is a unique gradient echo sequence that provides a signal acquisition with long TE and short TR by skipping the data acquisition during the first TR (16), which provides a rapid and sensitive acquisition for magnetic susceptibilities in the brain tissues including venous structures (17). Next, DSC-MRI scans were acquired for patients with acute ischemic stroke using a single shot T2^*^-weighted gradient EPI with the following parameters: TE = 40 ms, TR = 1,600 ms, flip angle = 75°, pixel size = 2.5 × 2.5 mm^2^, slice thickness = 4 mm, 25 slices, and FOV = 224 × 224 mm^2^. Dynamic scans covering the whole brain were subsequently performed to determine the affected volumes during a bolus injection: dynamic temporal resolution = 7.7 s, 10 pre-contrast scans, and 40 post-contrast scans. The contrast media (gadoterate meglumine at 1.0 mmol/mL to achieve 0.1 mmol/kg; range: 5–10 mmol per person) was injected via the antecubital vein at a rate of 3 mL/s using a power injector, which was followed by a bolus injection of 50 mL saline solution.

### QSM Reconstruction

The QSM was generated from the PRESTO sequence using our in-house scripts with MATLAB R2019b (MathWorks Inc., Natick, MA) (16). The phase images obtained using the PRESTO sequence were unwrapped using a Laplacian-based method to extract the total field (18). The tissue local field was calculated by the sophisticated harmonic artifact reduction for phase data with varying kernel sizes followed by the Laplacian boundary value method (19, 20). To minimize radio-frequency transmit-related phase offset (21), the local field map was corrected by fitting a three-dimensional polynomial function of the fourth-order to the resultant local field map (22). The final susceptibility map was reconstructed from the resultant local field map using improved sparse equations and the least squares method (23).

### OEF Calculation

The OEF maps were generated from the QSM images according to previously reported methods (13, 14). A venous mask was created by combining two approaches. A three-dimensional Gaussian high-pass filter (filter size: 5 × 5 × 5 voxels) was applied to the susceptibility map. To extract the small venous structures, a threshold value of mean + SD in the brain mask was applied to the high-pass filtered susceptibility map, based on the concept that venous susceptibility would be greater than brain parenchymal susceptibility, given the elevated deoxygenated hemoglobin concentration. Next, a fixed threshold of 0.03 ppm, which had been experimentally defined (24), was applied to the susceptibility map. The large structures such as the deep nuclei, hemosiderin deposition, dural sinuses, and large venous structures, were erased based on a volume of >1000 mm^3^ via morphological processing. The binary venous vessel masks extracted using the two approaches were multiplied to generate the venous mask. The susceptibility difference between the vein and surrounding brain tissue (Δχ) was expressed using the following equation [1]:


(1) $$\begin{array}{r} \Delta \chi =\Delta \chi_{do}\times Hct\times \left(1-Y_{v}\right)\times \frac{1}{P_{v}} \end{array}$$

where Δχ_*do*_ (0.18 ppm) is the difference in susceptibility per unit of hematocrit between fully deoxygenated and fully oxygenated blood (25), *Hct* is each subject's hematocrit at admission, *Y*_*v*_ is the venous oxygen saturation, and *P*_*v*_ (~4.1) is a correction factor for the partial volume effects that was defined based on the simulated calculation (13). The OEF value is defined as (*Y*_*a*_–*Y*_*v*_)/*Y*_*a*_, where *Y*_*a*_ is the arterial oxygen saturation and can be estimated as 1–*Y*_*v*_ under usual conditions in which *Y*_*a*_ is ~100% (26). Thus, the OEF value can be calculated using the following equation [2]:


(2) $$\begin{array}{r} OEF=\frac{\;\Delta \chi \times P_{v}}{\Delta \chi_{do}\times Hct} \end{array}$$

### DWI-PWI Mismatch

**Figure 1:**
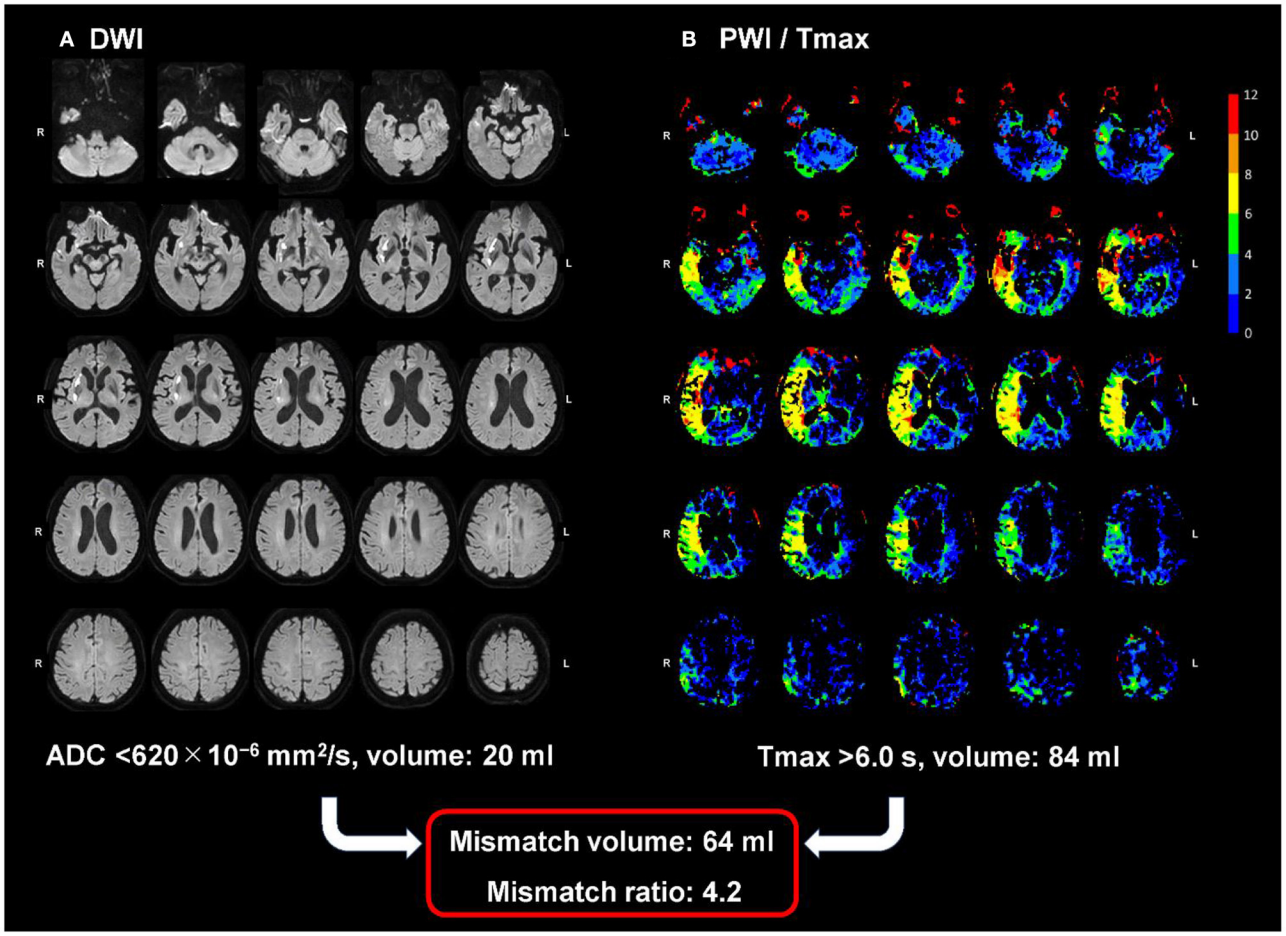
A representative case with Target Mismatch. The maps show DWI **(A)** and PWI/Tmax **(B)** with the right middle cerebral artery occlusion 90 min after symptom onset. The infarction core volume derived from DWI (apparent diffusion coefficient <620 × 10–6 mm^2^/s) is 20 ml and the ischemic penumbra volume derived from PWI (Tmax of >6 s) is 84 mL, indicating a mismatch ratio of 4.2 and a good candidate for reperfusion therapy. DWI, diffusion-weighted imaging; PWI, perfusion-weighted imaging.

The OEF values in acute ischemic stroke change dynamically based on the local tissue properties (27). Identification of ischemic penumbral tissues during the acute phase has been performed using the DWI-PWI mismatch concept (7–9). In this context, infarct core is defined as the irreversibly damaged region where the DWI-derived apparent diffusion coefficient is <620 × 10^−6^ mm^2^/s (28). After excluding the infarct core, the ischemic penumbra was defined as the region with a Tmax of >6 s, which is derived from DSC-PWI (29). Tmax is the time to maximum of the tissue residue function, which is generated by deconvolution of the tissue concentration-time curve with use of an arterial input function from the contralateral middle cerebral artery (30). The DWI and PWI images were imported into a freely available software (Perfusion Mismatch Analyzer, version 5.2) (31), which performs automatic measurements at regions of interest (32). The DWI-PWI mismatch is generally defined as the ratio of the ischemic penumbra volume to the infarct core volume, and the therapeutic target is identified at areas with a mismatch ratio of ≥1.8 (33). In the present study, patients with acute ischemic stroke were grouped according to mismatch ratios of ≥1.8 and <1.8 (i.e., with or without Target Mismatch) (33). Figure 1 shows a representative case with Target Mismatch based on the results from the Perfusion Mismatch Analyzer.

### OEF Measurement

Using Statistical Parametric Mapping 12 software (Welcome Department of Imaging Neuroscience, University College London, UK), the PRESTO-OEF and DSC-MRI images were coregistered to the Diffeomorphic Anatomical Registration Through Exponentiated Lie Algebra anatomical template (34), which was generated using the magnitude images from the PRESTO sequence. After normalization to the Montreal Neurological Institute space, the mean OEF values were measured for the penumbra (a DSC-PWI-derived Tmax of >6 s) and contralateral regions using the ITK-SNAP semiautomatic segmentation tool (University of Pennsylvania, Philadelphia, USA; www.itksnap.org) (35). The interhemispheric OEF ratios were also calculated between the penumbra and contralateral regions. The normal range for the OEF value was defined using the mean and SD values for the anterior circulation territory from the 21 healthy controls. The cut-off for an increased OEF value was defined as the mean + 2SD values from the healthy controls. Using that cut-off value, the increased OEF volumes were measured for the affected/contralateral hemisphere in patients with acute ischemic stroke.

### Statistical Analysis

Continuous variables were expressed as mean ± SD or median (interquartile range), based on the normality of data distribution, which was assessed using the Shapiro-Wilk test. Clinical characteristics, neuroimaging features, and outcomes were compared between the patients with acute ischemic stroke, with and without Target Mismatch, and the healthy controls, using the Kruskal-Wallis test or Fisher's exact test with *post-hoc* Bonferroni correction for multiple comparisons. Significance was set at a Bonferroni-corrected *P* value of <0.05.

The paired *t*-test was used to compare the OEF values for the penumbra and contralateral regions in patients with acute ischemic stroke. The Wilcoxon signed rank test was used to compare the increased OEF volumes for the affected hemisphere and the contralateral hemisphere in patients with acute ischemic stroke. Significance was set at a *P* value of < 0.05. Correlation analyses using Spearman's rank-order correlation coefficient and linear regression were also performed among patients with acute ischemic stroke to assess the relationships between the increased OEF volumes for the affected hemisphere (measured using the QSM-derived OEF map), the penumbral tissue volumes (volumes with a DSC-PWI-derived Tmax of >6 s), and the follow-up clinicoradiological findings (follow-up fluid-attenuated inversion recovery volumes at 30 days [i.e., the final infarction tissue volumes], the difference between the penumbral tissue volumes by perfusion imaging at admission and the follow-up fluid-attenuated inversion recovery volumes at 30 days [i.e., the salvaged penumbral tissue volumes], and the 30-day change in the NIHSS scores). Note that the salvaged penumbral tissue was defined as a region of the brain with formerly delayed perfusion and not infarcted (9). Dice similarity coefficients were calculated to measure the overlap fractions between penumbra volumes detected by the DSC and OEF methods. Finally, a receiver operating characteristic (ROC) curve analysis using the QSM-derived OEF map was performed to distinguish patients with and without Target Mismatch.

## Result

### Subject Characteristics

Table 1 summarizes the clinical characteristics, neuroimaging features, and outcomes. No significant inter-group differences were observed in terms of age, sex, premorbid modified Rankin Scale, or NIHSS score at arrival. The median time from stroke onset to MRI was shorter for patients with acute ischemic stroke and Target Mismatch (154 min [interquartile range: 114–278 min]) than for patients with acute ischemic stroke but no Target Mismatch (496 min [interquartile range: 198–810 min]). Among 11 patients with Target Mismatch, 7 patients underwent reperfusion therapy (intravenous recombinant tissue-type plasminogen activator and/or mechanical thrombectomy).” The occluded vessels included 7 internal carotid arteries, 3 anterior cerebral arteries, and 11 middle cerebral arteries. There were no significant differences between the patients with and without Target Mismatch (aside from the mismatch ratio) in terms of their neuroimaging features, treatments, and outcomes.

**Table 1:** Clinical characteristics, neuroimaging features, and outcomes.

	**Acute ischemic stroke patients**			
**Variables**	**With target**	**Without target**	**Controls**	***P* value**
	**mismatch (*n* = 11)**	**mismatch (*n* = 10)**	**(*n* = 21)**	
*Clinical characteristics*				
Age, years	75.2 ± 7.6	77.0 ± 7.5	75.3 ± 7.8	0.827
Women	5 (45.4)	4 (40.0)	10 (47.6)	0.924
Premorbid mRS	1 (0–2)	1 (0–2)	0 (0–0)	0.793
NIHSS score at arrival	12 (7–21)	13 (8–19)	NA	0.802
Time from stroke onset, min	154 (114–278)	496 (198–810)	NA	0.008
*Occluded vessels*				
Internal carotid artery	3 (27.3)	4 (40.0)	NA	0.537
Anterior cerebral artery	2 (18.2)	1 (10.0)	NA	0.593
Middle cerebral artery	6 (54.5)	5 (60.0)	NA	0.835
*Tissue properties*				
ADC (<620 × 10^−6^ mm^2^/s), ml	8.6 (7.1–22.0)	13.5 (5.1–26.6)	NA	0.860
Tmax (>6 s), ml	22.6 (14.9–−52.1)	16.3 (7.4–39.1)	NA	0.439
Mismatch volume, ml	11.9 (7.8–23.5)	3.6 (2.2–12.5)	NA	0.068
Mismatch ratio	2.4 (1.9–2.8)	1.4 (1.2–1.5)	NA	<0.001
*Treatments and outcomes*				
Intravenous rtPA	5 (45.5)	3 (30.0)	NA	0.466
Mechanical thrombectomy	4 (36.4)	2 (20.0)	NA	0.696
FLAIR at 30 days, ml	10.5 (9.9–28.0)	29.2 (10.2–42.2)	NA	0.290
mRS at 30 days	3 (2–4)	4 (2–4)	NA	0.123
NIHSS score at 30 days	8 (4–10)	12 (4–14)	NA	0.148

*Data are mean ± standard deviation, median (interquartile range), or number (%). ADC, apparent diffusion coefficient; FLAIR, fluid-attenuated inversion recovery; mRS, modified Rankin Scale; NA, not applicable; NIHSS, National Institutes of Health Stroke Scale; rtPA, recombinant tissue-type plasminogen activator*.

### Representative Images

**Figure 2:**
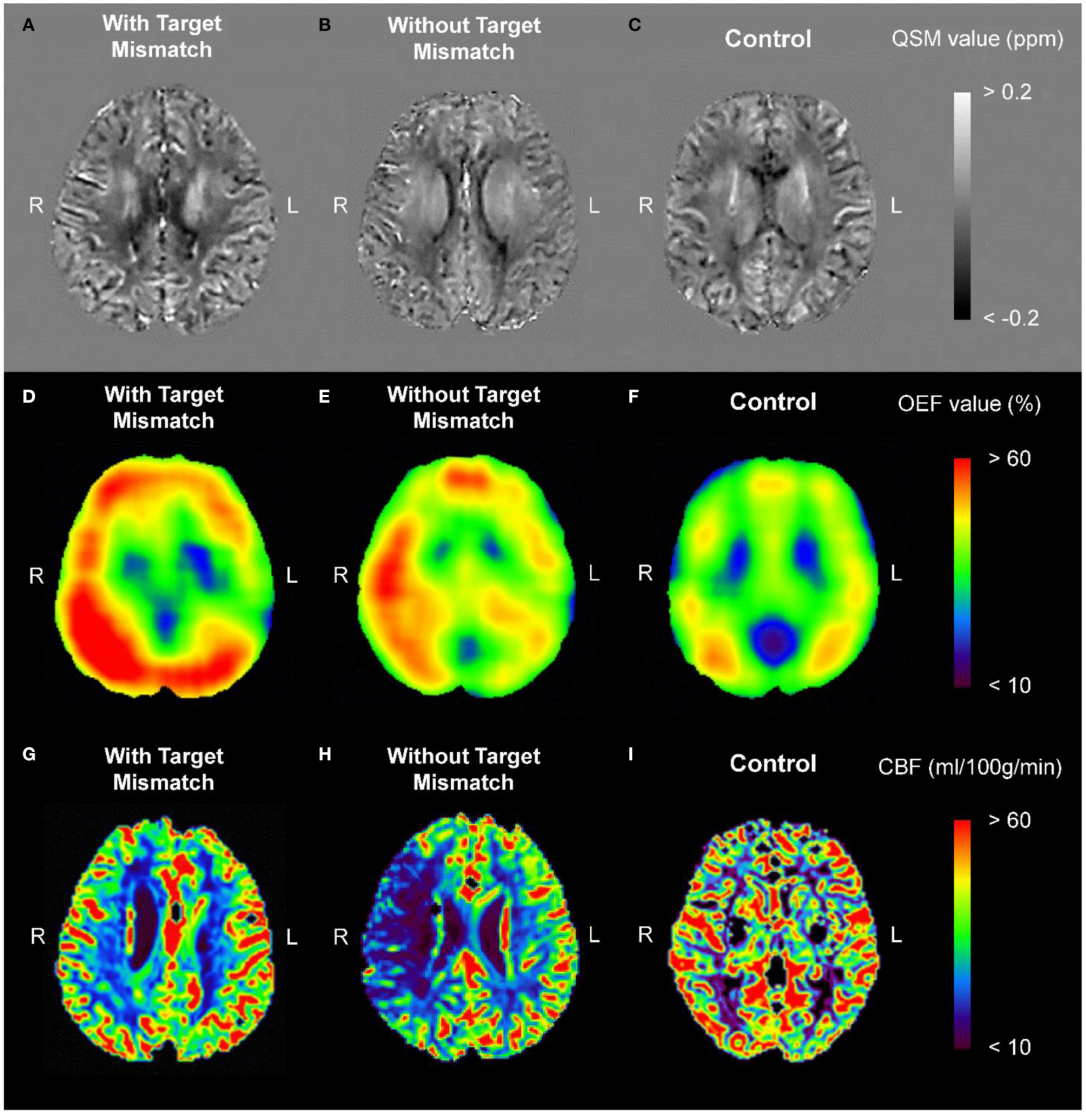
Representative images from PRESTO-QSM **(A–C)** and the corresponding OEF **(D–F)** and CBF **(G–I)** maps for a patient with acute ischemic stroke and Target Mismatch [right internal carotid artery occlusion; **(A,D,G)**], a patient with acute ischemic stroke but no Target Mismatch [right middle cerebral artery stenosis; **(B,E,H)**], and a healthy control **(C,F,I)**. CBF, cerebral blood flow; OEF, oxygen extraction fraction; PRESTO, principles of echo shifting with a train of observations; QSM, quantitative susceptibility mapping.

Figure 2 shows representative images from PRESTO-QSM and the corresponding OEF and cerebral blood flow maps for a patient with acute ischemic stroke and Target Mismatch (right internal carotid artery occlusion), a patient with acute ischemic stroke but no Target Mismatch (right middle cerebral artery stenosis), and a healthy control.

### OEF Measurements

Table 2 summarizes the OEF measurements. The mean normal OEF value was 44.7 ± 3.4%, which was defined using the anterior circulation territory OEF values from the 21 healthy controls. Therefore, the upper limit of normal (mean + 2SD) for the OEF value was defined as 51.5%, and values of >51.5% were considered increased. The OEF values for the penumbra region (a DSC-PWI-derived Tmax of >6 s, the “affected region” in Table 2) were higher than those in the contralateral region for patients with acute ischemic stroke (mean difference: 6.1, 95% CI: 5.0 to 7.2, *P* < 0.001). The increased OEF volumes in the affected hemisphere (volumes with OEF values of >51.5% derived from the QSM-based OEF map, the “affected side” in Table 2) were also higher than those in the contralateral hemisphere for patients with acute ischemic stroke (mean difference: 9.5, 95% CI: 3.9 to 15.1, *P* < 0.001). There were no significant differences between the patients with and without Target Mismatch in the OEF values, interhemispheric OEF ratios, and increased OEF volumes.

**Table 2:** OEF measurements.

	**Regions of interest**	**OEF value, %**	**Interhemispheric ratio**	**Increased OEF volume, ml**
Healthy controls (*n* = 21)	Anterior circulated territory	44.7 ± 3.4	NA	0.9 (0.0–1.9)
Acute ischemic stroke patients (*n* = 21)	Affected region/side	50.9 ± 4.8	1.14 ± 0.08	11.0 (3.0–23.5)
	Contralateral region/side	44.8 ± 3.7	NA	1.4 (0.5–2.0)
With Target Mismatch (*n* = 11)	Affected region/side	51.3 ± 4.9	1.15 ± 0.06	12.8 (3.3–23.0)
	Contralateral region/side	44.5 ± 3.5	NA	1.2 (0.4–1.6)
Without Target Mismatch (*n* = 10)	Affected region/side	50.5 ± 4.7	1.12 ± 0.05	9.7 (2.9–31.1)
	Contralateral region/side	45.0 ± 3.9	NA	1.5 (0.5–2.0)

*Data are mean ± standard deviation or median (interquartile range). NA, not applicable; OEF, oxygen extraction fraction*.

### Correlation Analyses

**Figure 3:**
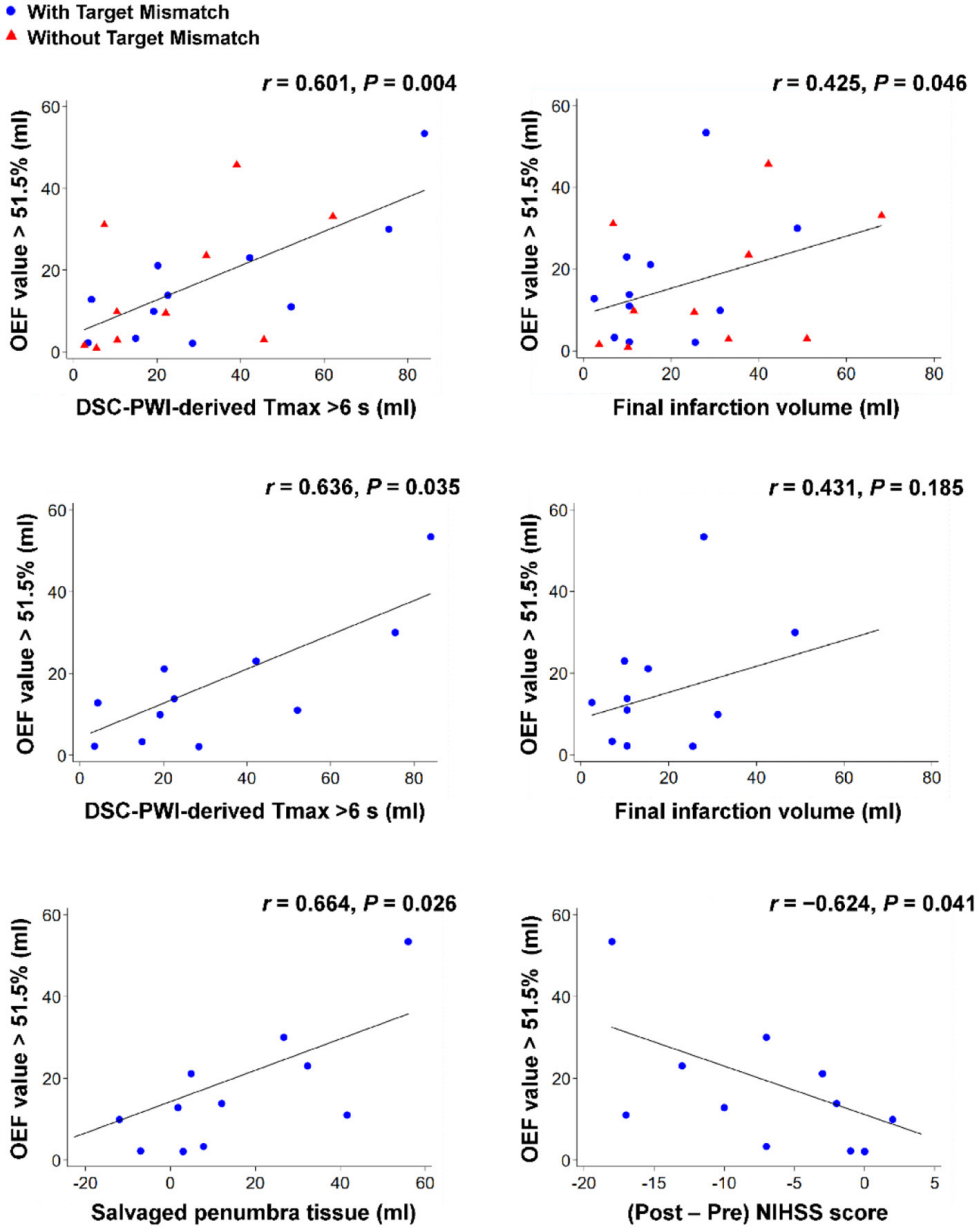
Overview of correlation analyses between the increased OEF volumes (volumes with OEF values of >51.5% derived from the QSM-based OEF map) in the affected hemisphere, the penumbral tissue volumes (volumes with a DSC-PWI-derived Tmax of >6 s), and the follow-up clinicoradiological findings (follow-up fluid-attenuated inversion recovery volumes at 30 days [i.e., the final infarction tissue volumes], the difference between the penumbral tissue volumes by perfusion imaging at admission and the follow-up fluid-attenuated inversion recovery volumes at 30 days [i.e., the salvaged penumbral tissue volumes], and the 30-day change in the NIHSS scores) for patients with acute ischemic stroke. Note that blue circles show patients with Target Mismatch and red triangles show patients without Target Mismatch. DSC, dynamic susceptibility contrast; NIHSS, National Institutes of Health Stroke Scale; OEF, oxygen extraction fraction; PWI, perfusion-weighted imaging.

Figure 3 shows the results of the correlation analyses for the relationships between the increased OEF volumes (volumes with OEF values of >51.5% derived from the QSM-based OEF map) in the affected hemisphere, the penumbral tissue volumes (volumes with a DSC-PWI-derived Tmax of >6 s), and the follow-up clinicoradiological findings in patients with acute ischemic stroke. In all patients, the increased OEF volumes were positively correlated with the penumbral tissue volumes (r = 0.601, 95% CI: 0.229 to 0.820; *P* = 0.004) and the final infarction tissue volumes (r = 0.425, 95% CI: 0.011 to 0.818; *P* = 0.046). In patients with Target Mismatch, the increased OEF volumes were positively correlated with the penumbral tissue volumes (r = 0.636, 95% CI: 0.059 to 0.895; *P* = 0.035) and the salvaged penumbral tissue volumes (r = 0.664, 95% CI: 0.106 to 0.904; *P* = 0.026) and inversely correlated with the 30-day change in the NIHSS scores (r = −0.624, 95% CI: −0.891 to −0.039; *P* = 0.041). None of the values were correlated in patients without Target Mismatch.

### Dice Similarity Coefficients

Overlapping fractions between penumbra volumes detected by the DSC and OEF methods were calculated as the Dice similarity coefficients (mean ± SD), showing high overlapping fractions for all the patients (0.724 ± 0.152), patients with Target Mismatch (0.781 ± 0.118), and patients without Target Mismatch (0.675 ± 0.186).

### ROC Curve Analysis

**Figure 4:**
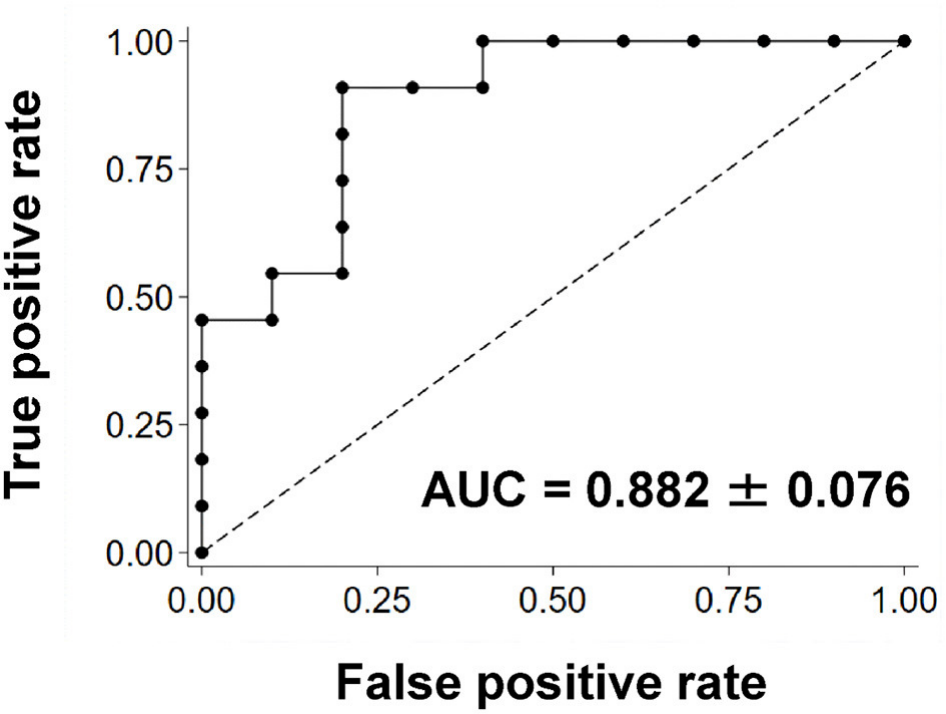
The ROC curve using the QSM-derived OEF map to distinguish patients with and without Target Mismatch. The area under the ROC curve was 0.882 ± 0.076. The sensitivity and specificity were 90.9 and 80.0%, respectively, when the cut-off of increased OEF value was set at ≥9.9. AUC, area under the curve; OEF, oxygen extraction fraction; QSM, quantitative susceptibility mapping; ROC, receiver operating characteristic.

Figure 4 shows the results of the ROC curve analysis using the QSM-derived OEF map to distinguish patients with and without Target Mismatch. The area under the ROC curve was 0.882 ± 0.076. The sensitivity and specificity were 90.9 and 80.0%, respectively, when the cut-off of increased OEF value was set at ≥9.9.

## Discussion

Using a rapid acquisition for whole-brain magnetic susceptibility by a conventional 3.0 T MRI system in patients with acute ischemic stroke, we revealed for the first time that the increased OEF volumes derived from the QSM-based OEF map were correlated with the ischemic penumbra volumes with a DSC-PWI-derived Tmax of >6 s. Furthermore, the mean anterior circulation territory OEF value from 21 healthy controls (44.7 ± 3.4%) was comparable to the ^15^O-PET-OEF value (1), which is the gold standard for penumbra detection. Consequently, the MRI-based OEF estimation could be useful for identifying ischemic penumbral tissues, and could be a non-invasive alternative to ^15^O-PET and DSC-MRI scans in an urgent care setting.

The penumbra has been defined as the area as ischemic tissues between the upper threshold of electrical failure and the lower threshold of energy and ion pump failure (36). The last four decades have witnessed the rapid development of clinical neuroimaging tools that are now increasingly applied in the acute stroke setting. The penumbra is considered as ischemic tissues that are potentially destined for infarction but are not irreversibly damaged and can be targeted for acute treatment (37). Reperfusion therapy for patients with acute ischemic stroke has been improved by the evolution of mechanical thrombectomy with or without intravenous recombinant tissue plasminogen activator (8, 9). Thus, it is critically important to predict the salvageable penumbral tissue volume in order to select appropriate acute phase treatments.

Various computed tomography- and/or MRI-based neuroimaging biomarkers correlate with clinical outcomes among patients with acute ischemic stroke, including the Alberta Stroke Program Early Computed Tomography Score (38), venous imaging (39), collateral flow status (40), and mismatch between the ischemic core and hypoperfusion volume (7–9). Recent studies have used a DWI-PWI volume mismatch ratio of ≥1.8 to identify therapeutic targets and revealed favorable treatment outcomes (9, 41), which we also applied in the present study of patients with acute ischemic stroke. However, the DSC-MRI protocol is contraindicated for patients with a history of renal impairment (42), which suggests that estimated glomerular filtration rate must be confirmed before perfusion imaging. Thus, the DSC-MRI preparation and scanning procedures last considerably longer than the procedures for conventional MRI. Regarding hematocrit value requirements, a standard value, such as hematocrit = 0.45, can be input instead of raw data for every subject if complete blood count data are unavailable (13, 14).

An appealing feature of the PRESTO-QSM approach is its non-invasive nature and ability to quantitatively estimate the OEF value in acute stroke using a conventional 3.0 T MRI system. While the ^15^O-PET-derived OEF value remains the gold standard for penumbra detection (6), the procedure is too complex for an urgent care setting, necessitating the need for a technique that identifies penumbral tissues accurately and more rapidly (27). Since the salvageable penumbra volume tends to decrease over time after stroke onset, we grouped the patients according to their DWI-PWI mismatch ratio to validate the MRI-based OEF estimation as a penumbra biomarker. This patient grouping aligned with our hypothesis that ischemic penumbra volumes determined using the DWI-PWI mismatch would be associated with increased OEF volumes derived from PRESTO-QSM.

The QSM-based OEF values reportedly exhibit a good agreement with the values from ^15^O-PET (13). Furthermore, QSM-based OEF maps have been applied clinically for patients with chronic cerebrovascular disease (14). The PRESTO sequence allows us to achieve rapid data acquisition with strong T2^*^-weighted contrast, using the echo-shifted pulse sequence (TE >TR) (17). The long TE increases sensitivity to the proton resonance frequency offset and the signal to noise ratio of the phase images (43). Since the echo-shifted pulse sequence uses gradient echo, it is more robust in terms of image quality (e.g., blurring and distortion) than the echo planar imaging sequence (43). The accuracy of QSM reconstruction using the PRESTO sequence was comparable to the spoiled gradient-recalled echo sequence with flow compensation in human brain experiments (16). The present study also revealed that OEF maps could be created using PRESTO-QSM in patients with acute ischemic stroke and that these maps could be used to clinically estimate the ischemic penumbra volume.

Other MRI-based methods that enable OEF extraction should be discussed. Using venular-targeted velocity-selective spin labeling, quantitative imaging of extraction of oxygen and tissue consumption is useful for mapping OEF (44). Asymmetrically prominent cortical veins are manifestations of increased OEF (45, 46). Moreover, combining analysis of QSM for phase and quantitative blood oxygenation level-dependent for magnitude, that is QQ algorithm developed by Cho et al. (47), enables OEF extraction directly from tissue voxels. The QQ-based OEF was compared with ^15^O-PET (48) and shown to be feasible to patients with ischemic stroke (49). Unfortunately, however, the QQ analysis could not have been applied to the current study because the PRESTO sequence was single-echo acquisition to reduce acquisition time, which is TR < TE, for an urgent care setting with maintaining the signal to noise ratio of phase signal (16).

Despite our small sample of patients with Target Mismatch, the increased OEF volumes were positively correlated with the penumbral tissue volumes and inversely correlated with the 30-day change in the NIHSS scores. However, these values were not correlated in the patients without Target Mismatch. In this context, the OEF values fluctuate within the tissues from the penumbra to the infarction core (50). A longitudinal evaluation of QSM-based OEF values in patients with acute ischemic stroke revealed that the increased OEF values of the penumbral tissues normalized within several days (15). Therefore, we defined the ischemic penumbral tissue volume as the volume with a Tmax of >6 s after excluding the volume of the ischemic core (29). An ongoing longitudinal study is also attempting to determine whether OEF changes, which are measured using the PRESTO-QSM-derived OEF map, are correlated with clinical outcomes after reperfusion therapy.

The present study has several limitations. It is important to exercise caution when interpreting magnetic susceptibility differences as deoxygenated hemoglobin-related changes. For example, increases in magnetic susceptibility values can be created by other substances, such as iron and aluminum (51), and the current QSM approaches are unable to identify the chemical configuration underlying the abnormal magnetostatic behaviors. However, we excluded higher paramagnetic and large structures during the venous mask creation. Although the prior works using QSM-based OEF extraction based on oxygenation of the draining veins used submillimeter resolutions (12–14), the current study algorithm (PRESTO-QSM-based OEF extraction) used iso-voxel resolution of 1.5 mm^3^. It is also important to note that using the PRESTO sequence without gradient moment nulling could generate artificial signal loss in the blood vessels. Moreover, phase images are easily affected by artifacts induced from blood flow around the vessels (16). These effects on the susceptibility map in and around the vessels are caused by the deconvolution process of QSM analysis, although the differences in phase values are considered subtle relative to the spoiled gradient-recalled echo sequence (52). Finally, the study's small sample size and limited follow-up period preclude conclusions regarding the ability of our strategy to predict favorable and/or poor long-term outcomes, or whether the therapeutic range for delayed stroke could be expanded beyond the established time frames.

## Conclusion

This observational cohort study revealed that the increased OEF volumes derived from the PRESTO-QSM-based OEF estimation were correlated with the penumbra volumes with a DSC-PWI-derived Tmax of >6 s in patients with acute ischemic stroke. The increased OEF volumes were also correlated with the 30-day follow-up radiological and clinical findings. These results highlight the potential of the MRI-based OEF map as a penumbra biomarker to guide treatment selection in acute ischemic stroke.

## Data Availability Statement

The raw data supporting the conclusions of this article will be made available by the authors, without undue reservation.

## Ethics Statement

The studies involving human participants were reviewed and approved by Institutional Review Board of Toyokawa City Hospital. The patients/participants provided their written informed consent to participate in this study.

## Author Contributions

YU: conceptualization, methodology, software, formal analysis, investigation, data curation, writing-original draft, and funding acquisition. HK: conceptualization, formal analysis, investigation, data curation, supervision, resources, writing-review & editing, and funding acquisition. HI, MO, HS, TK, TU, KT, YK, and KY: data curation and investigation. KK and NM: supervision and writing-review & editing. All authors contributed to the article and approved the submitted version.

## Funding

This work was supported by Grants-in-Aid for Young Scientists (19K17148 to YU, 17K15805 to HK) from Japan Society for the Promotion of Science.

## Conflict of Interest

The authors declare that the research was conducted in the absence of any commercial or financial relationships that could be construed as a potential conflict of interest.

## Publisher's Note

All claims expressed in this article are solely those of the authors and do not necessarily represent those of their affiliated organizations, or those of the publisher, the editors and the reviewers. Any product that may be evaluated in this article, or claim that may be made by its manufacturer, is not guaranteed or endorsed by the publisher.
